# The Diagnosis and Treatment of Fractures

**Published:** 1907-04-13

**Authors:** 


### Points in Surgery.

### THE DIAGNOSIS AND TREATMENT OF FRACTURES.

### The Uses of the Röntgen Rays.

The practitioner will do well to keep in close touch with the advances made in the surgical treatment of fractures, not only for the sake of his patients, but also for the sake of his own reputation. Since the application of Röntgen rays as a guidance in the diagnosis and treatment of injuries to bones in the last ten years, and the introduction of massage, more advances are being made in our knowledge and treatment of fractures than was ever before possible. The practitioner cannot be regarded as fully equipped without the *x*-ray apparatus. It is suggested that, for mutual protection, a number of practitioners in the smaller towns should jointly set up a complete installation. The general public is becoming familiar with the application of *x*-rays. It realises that to see the position of broken bones in their relation to one another must be a guidance to the surgeon. Though in some cases and in certain fractures the methods of treatment which have long been recognised have undergone no material modification, yet it is reasonable and right that an examination by the rays should be made whenever possible.

Many important cases of litigation have arisen since the introduction and application of Röntgen's discovery. In more than one instance the defendant practitioner has failed to convince the adjudicators that all reasonable skill had been applied. Had a radiograph been available it should not have been difficult to show the skill which had been brought to bear, or that it had been reasonable. But it must also be borne in mind that a radiograph requires skilled interpretation.

The popular notion that by the employment of *x*-rays it is possible for the surgeon to set the fragments in perfect apposition, which can be portrayed, should be explained away. Some linear imperfection is inevitable in the most perfectly reduced fracture, and this is most readily explained by the fact that the radiograph is not a photograph of the object exposed to the *x*-rays, but is in reality a representation of its shadow. The rays diverge from the terminals in the vacuum tube, casting a shadow on the sensitive plate, a shadow which is greater than the object it represents.

Skilfully to interpret a radiograph, it is necessary to know the relative positions of, first, the point from which the rays emanate; secondly, of the negative plate; and, thirdly, of the intervening object casting its shadow on the plate. For a radiograph is a *silhouette*. The closer the object to the plate the more perfect the silhouette. It is for these reasons that to rely implicitly on the evidence which a radiograph seems to afford would lead into serious error. To understand, therefore, the meaning of the shadows we must compare shadows thrown from more than one direction. In the case, say, of fractures of both bones of the forearm, the limb should be examined from the front and from the side.

### A Case of Fracture of the Forearm.

The following instance may be cited. A carpenter received a blow from a brick on his right forearm: he was at once seen by a medical man, who diagnosed by manipulation fracture of both bones about the middle of the shaft. The limb was put up with an internal angular splint, fixing the elbow and the wrist with the forearm, in a position midway between pronation and supination. Next day the patient went to another medical man, who was asked to assume charge of the case. This he refused to do unless the patient first had an *x*-ray examination. This was made without taking off the splints, and it was found that the radius and ulna were transversely fractured at the same level about the middle of the shaft. The fragments slightly overlapped, but what was more important, the upper end of the ulna was in apposition with the lower end of the radius, and had the bones been allowed to remain in this position all four fragments would no doubt have firmly consolidated abolishing all power of pronation and supination of the forearm, so that the patient would have been deprived of his means of livelihood.

The matter was explained to the patient, and he was advised to have a general anæsthetic to permit the correct readjustment of the fragments, the practitioner objecting to take charge of the case unless this was done. An anæsthetic was given, the splints were removed, and the limb was put up with an anterior elbow splint, with the elbow flexed, and the wrist fully supinated. Radiographs were taken in two directions, and it was seen that almost perfect apposition had been produced. The limb was treated by massage and passive movement, and in a few weeks complete function was restored. There is little doubt that without the use of *x*-rays in this case the patient would have lost the proper use of his forearm, and the practitioners might have been involved in a troublesome and expensive lawsuit.

### Fallacies to be Avoided.

By use of the fluorescent screen in the first place a clear impression may be obtained of the fracture, so that one may determine from what point of view a radiograph may be taken to give the best results.

Certain fallacies must be guarded against; for instance, epiphysial cartilages in young subjects have been mistaken for lines of cleavage due to fracture. In the injuries in the neighbourhood of joints, *e.g.*, the elbow, it is necessary to remember that unossified epiphyses throw no shadows. The ossifying centre of the epiphysis may be mistaken for a displaced fragment of bone. Remember, too, the dates of appearance of the ossific centres in the epiphyses. At the lower end of the humerus, this is especially important, in order to interpret the radiograph aright. The capitellum begins to cast a shadow about the third year, the internal condyle is visible about the fifth year, when that for the capitellum is much larger. That for the trochlear appears about the tenth year, and the small centre for the external condyle casts a shadow about the fifteenth year, when the other three centres are fused in one shadow.

